# Efficient induction of differentiation and growth inhibition in IDH1 mutant glioma cells by the DNMT Inhibitor Decitabine

**DOI:** 10.18632/oncotarget.1412

**Published:** 2013-09-16

**Authors:** Sevin Turcan, Armida W. M. Fabius, Alexandra Borodovsky, Alicia Pedraza, Cameron Brennan, Jason Huse, Agnes Viale, Gregory J. Riggins, Timothy A. Chan

**Affiliations:** ^1^ Human Oncology and Pathogenesis Program, Memorial Sloan-Kettering Cancer Center, New York, NY, USA; ^2^ Department of Neurosurgery, School of Medicine, Johns Hopkins University, Baltimore, MD; ^3^ Genomics Core, Memorial Sloan-Kettering Cancer Center, New York, New York, USA; ^4^ Dept. of Radiation Oncology, Memorial Sloan-Kettering Cancer Center, New York, New York, USA; ^5^ Brain Tumor Center, Memorial Sloan-Kettering Cancer Center, New York, New York, USA

**Keywords:** glioma, isocitrate dehydrogenase, methylation, epigenetics, treatment

## Abstract

Mutation in the *IDH1* or *IDH2* genes occurs frequently in gliomas and other human malignancies. In intermediate grade gliomas, IDH1 mutation is found in over 70% of tumors. These mutations impart the mutant IDH enzyme with a neomorphic activity – the ability to synthesize 2-hydroxyglutarate (2-HG). This ability leads to a reprogramming of chromatin state, a block in differentiation, and the establishment of the glioma hypermethylator phenotype (G-CIMP). It has been hypothesized but not proven that the extensive DNA methylation that occurs in G-CIMP tumors helps maintain and “lock in” glioma cancer cells in a dedifferentiated state. Here, we tested this hypothesis by treating patient derived IDH1 mutant glioma initiating cells (GIC) with non-cytotoxic, epigenetically targeted doses of the DNMT inhibitor decitabine. Global methylome analysis of treated IDH1 mutant GICs showed that DAC treatment resulted in reversal of DNA methylation marks induced by IDH and the re-expression of genes associated with differentiation. Accordingly, treatment of IDH1 mutant glioma cells resulted in a dramatic loss of stem-like properties and efficient adoption of markers of differentiation, effects not seen in decitabine treated IDH wild-type GICs. Induction of differentiation was much more efficient than that seen following treatment with a specific inhibitor of mutant IDH enzyme (Agios). Decitabine also decreased replicative potential and tumor growth *in vivo*. Reexpression of polycomb regulated genes accompanied these DAC-induced phenotypes. In total, our data indicates that targeting the pathologic DNA methylation in IDH mutant cells can reverse mutant IDH induced hypermethylation and block in differentiation and promote tumor control. These findings have substantial impact for exploring new treatment strategies for patients with IDH mutant gliomas.

## INTRODUCTION

Mutations in the metabolic enzyme isocitrate dehydrogenase 1 (IDH1) are found in >70% intermediate grade gliomas [1, 2], a disease which eventually progresses to high-grade glioma within 10 years. These mutations confer gain-of-function activity that allows the production of (*R*)-2-hydroxyglutarate (2-HG) [3, 4], a metabolite that is normally present at trace levels. Accumulation of 2-HG competitively inhibits various α-ketoglutarate-dependent dioxygenases [5]. IDH1 mutation functions by directly remodeling the epigenome to establish the glioma CpG island methylator phenotype (G-CIMP), inhibiting histone lysine demethylases and causing a block to cellular differentiation [6-9].

It has been hypothesized that the extensive DNA methylation that occurs in G-CIMP tumors maintains glioma cancer cells in a dedifferentiated state. The aberrant gene expression profile activated by mutant IDH1 confers a block to differentiation causing the malignant expansion of tumor-initiating cells with capacity to self-renew [6, 8]. These findings raise the possibility that erasing the aberrantly hypermethylated marks may reverse the differentiation block induced by mutant IDH1. To explore this therapeutic possibility, we used the DNA demethylating agent, decitabine, a Food and Drug Administration (FDA) approved drug, to treat patient derived glioma tumor cells. We analyzed the effects of decitabine on both GICs with and without an endogeneous IDH1 mutation. IDH1-mutant GIC has been described previously [10].

The cytosine analogue 5-aza-2'-deoxycytidine (decitabine, DAC) is a hypomethylating agent used as a treatment for myelodysplastic syndrome. DAC exerts its effect by depletion and degradation of the maintenance DNA methyltransferase, DNMT1. Exposure to DNA demethylating agents is associated with altered hematopoietic differentiation and results in terminal differentiation of leukemia cells [11, 12]. Further, DAC has the ability to cross the blood-brain barrier – the level of DAC attained in the cerebrospinal fluid can reach as high as half of its plasma concentration after a continuous intravenous infusion [13], making this drug an attractive therapeutic option for the management of gliomas. Recent studies have shown the efficacy of using low, epigenetically targeted doses of DNA demethylating agents in producing an antitumor memory response in both leukemic and epithelial tumors, including inhibition of subpopulations of cancer stem-cell like cells [14].

Although the impact of targeting the mutant enzyme with an IDH1 specific inhibitor has been evaluated [10], the effect was modest and did not lead to tumor regression. The efficacy of using DNA demethylating agents to treat mutant IDH1 expressing glioma cells has yet to be tested. Our results indicate that transient low doses of decitabine increases expression of genes associated with glial-astrocytic differentiation and induces differentiation in patient-derived IDH1-mutant tumor spheres. These findings begin to explore the efficacy of using an FDA approved drug in the management of IDH mutant gliomas.

## RESULTS

### DAC induces differentiation of mutant IDH1 expressing glioma cells

**Figure 1:**
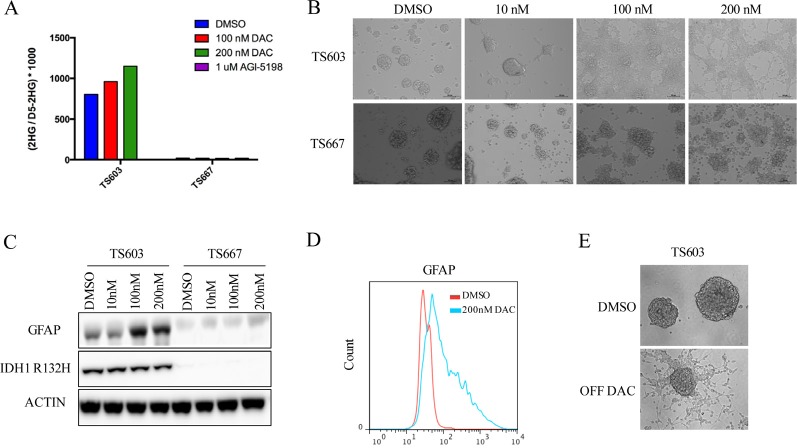
Decitabine efficiently induces differentiation in IDH1 mutant patient derived glioma initiating cells A, DAC does not decrease 2HG levels. TS603 (IDH1 mutant) and TS667 (IDH wild-type) cells were treated as shown. For comparison, the mutant IDH1 inhibitor AGI-5198 was used in parallel, which dramatically lowered 2-HG levels in the TS603 line. B, DAC induces a differentiated morphology. Cells were treated with the indicated concentrations of DAC and bright-field images were taken at 10X magnification. At 200nM DAC, TS603 cells were adherent while the TS667 cells remained non-adherent spheres. C, DAC induces GFAP in TS603 cells but not in TS667 cells. Results from western blot with the indicated antibodies are shown. D, Flow cytometry results showing induction of GFAP protein levels in DAC treated IDH1 mutant TS603 cells. E, TS603 cells retain an adherent phenotype after withdrawal of DAC. Cells were treated with 200nM DAC for 7 days and then drug was removed and the cells were cultured for 3 weeks.

To study the effect of DAC on mutant IDH1 expressing gliomas, we utilized glioma tumor spheres that carry an endogenous heterozygous R132H mutation (TS603). These cells were derived from a patient with WHO grade III anaplastic oligodendroglioma and harbor a co-deletion of 1p and 19q. TS603 exhibits the G-CIMP phenotype and produces high 2HG levels *in vitro* [10]. As a control, we used the IDH wild-type oligogendroglioma tumor sphere line TS667. We used DAC at a nanomolar range (10, 100 and 200 nM) to treat TS603 and TS667 glioma cells. These levels are non-cytotoxic [14]. 2-HG levels were unchanged in pellets of TS603 glioma cells after 7 days of treatment (Fig. 1A). Strikingly, 3 days of continuous exposure to DAC led to dramatic changes in the morphology of TS603 cells. At the 200 nM dose, treated TS603 cells exhibited a differentiated morphology and became adherent (Fig. 1B). Furthermore, the differentiation phenotype was dose dependent, and was observed even at 10 nM DAC where some cells grew as adherent spheres with a few differentiated cells in between spheres (Fig. 1B). Vehicle treated TS603 and TS667 cells and DAC treated TS667 cells continued to grow strictly as non-adherent spheres in culture and did not differentiate, suggesting that the differentiation phenotype is IDH1 mutant specific.

Next, we assessed protein levels of GFAP, a marker for glial differentiation. GFAP protein expression was markedly increased in TS603 cells after 3-day treatment with 100 or 200 nM DAC compared to vehicle treated cells (Fig. 1C, D). We did not observe any increase in GFAP expression in IDH wild-type TS667 cells.

We sought to determine whether transient treatment with DAC resulted in a “memory” type response that has recently been shown for transient low doses of DNA demethylating agents in hematological and epithelial tumors [14]. To test this hypothesis, we treated TS603 for 7 days with 200 nM DAC, followed by drug withdrawal and culture in drug-free media for 3 weeks. While DNMT1 protein levels quickly recovered, the differentiation phenotype was maintained (but did reverse slowly) and transiently treated cells continued to grow as adherent cells (Fig. 1E).

Taken together, these results indicate that decitabine is able to efficiently reverse the differentiation block induced by mutant IDH1.

### Low dose DAC markedly impairs growth of mutant IDH1 expressing glioma cells

**Figure 2:**
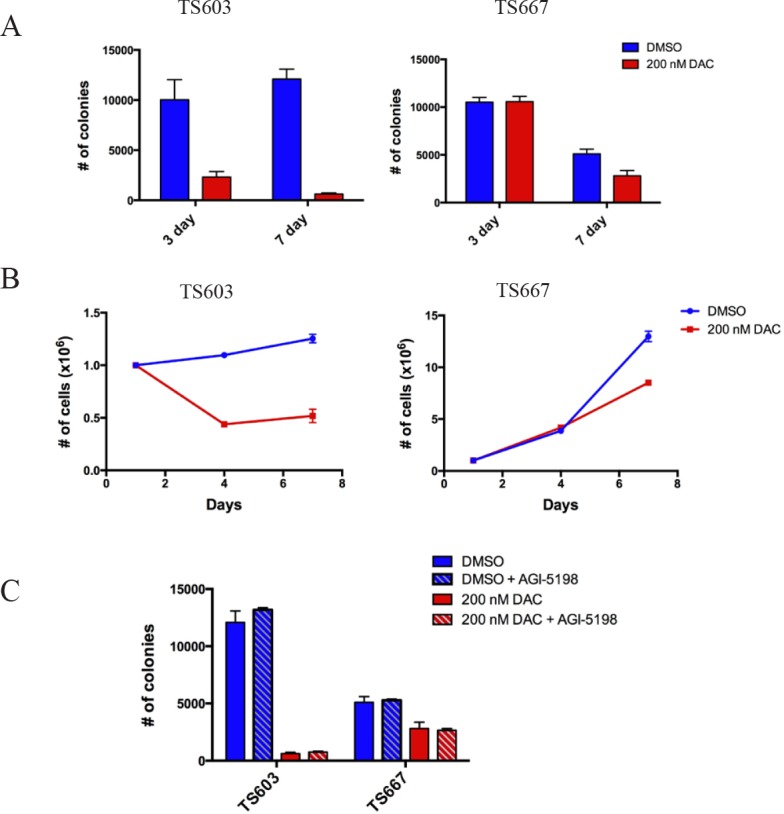
Low dose decitabine impairs growth potential*in vitro* and is superior to AGI-5198 in reducing proliferative capacity A, Results from anchorage-independent growth assays using soft agar. Effect of DAC on IDH mutant TS603 and IDH wild-type TS667 cells. Cells were treated with 200nM DAC. All experiments done in triplicate. Data shown is mean +/− 1 standard deviation. B, Cell growth curves showing that DAC treatment (200nM) decreases the proliferation of TS603. C, Effects of DAC (200nM) + AGI-5198 (1uM). The anti-proliferative effects observed with DAC alone were the same as with the combination. The growth suppressive effect was due entirely to DAC and not AGI-5198.

We found that both 3- and 7- day exposure to 200 nM DAC led to a significant decrease in colony formation ability of TS603 cells in soft agar, with >90% reduction in colony formation ability occurring after 7-day exposure (Fig. 2A, left panel). In addition, cell growth was also suppressed by 60% in mutant IDH1 expressing TS603 after 3- and 7- days of 200 nM DAC treatment (Fig. 2B, left panel). Although potent in the IDH mutant cells, the decrease in tumorigenicity was not entirely specific to TS603 cells. TS667 cells also showed decreased colony formation ability and cell growth, although the affect was not as dramatic and only occurred after 7 days of treatment with 200 nM DAC. (Fig. 2A-B, right panels)

Next, we tested the efficacy of combining DAC with AGI-5198, a mutant IDH1 specific inhibitor. AGI-5198 is highly selective for R132H mutation and under near complete 2HG inhibition induces modest expression of differentiation-associated genes. This is accompanied by decreased proliferation of IDH1-mutant glioma cells, without causing significant changes in genome-wide DNA methylation levels [10]. The effects of AGI-5198 were subtle and tumor regression never occurred. Therefore, we hypothesized that pre-treatment with 200 nM DAC may further ‘sensitize’ the IDH1-mutant glioma cells to the therapeutic effects of AGI-5198. TS603 and TS667 cells were treated for 7 days with 200 nM DAC, followed by DAC removal and plating in soft agar, at which point the cells were either treated with 1μM of AGI-5198 or DMSO continuously for 3 weeks. Pre-treatment with DAC did not allow the TS603 cells to respond more effectively to AGI-5198 (Fig. 2C). The numbers of colonies in DAC pretreated cells were similar between inhibitor and DMSO treated TS603 glioma cells, indicating that the dramatic reduction in anchorange-independent growth can be attributed to DAC alone. AGI-5198 did not cause additional loss of anchorage-independent growth.

**Figure 3:**
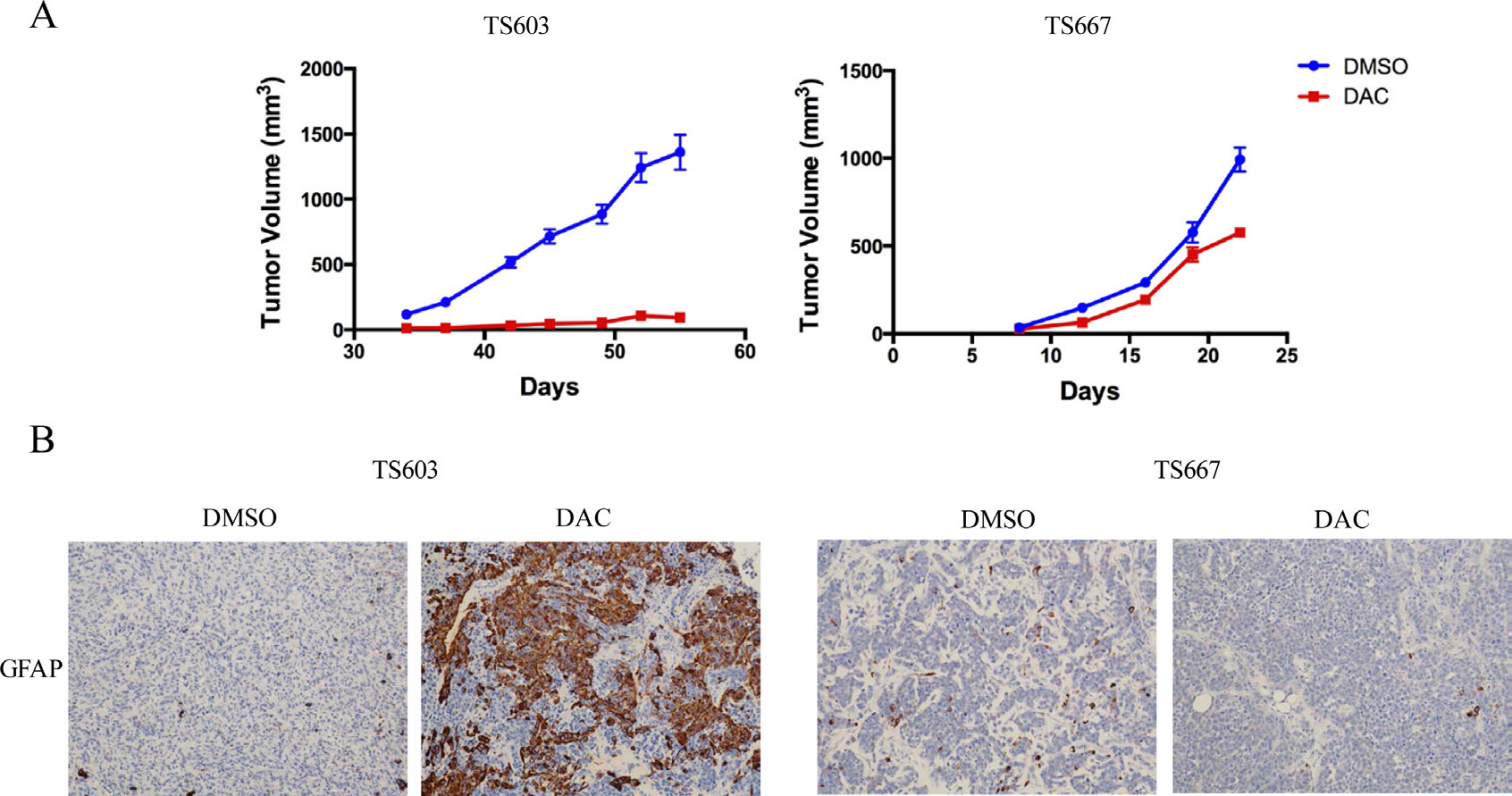
DAC suppresses growth and promotes differentiation of IDH mutant glioma cells A, Xenograft assays showing that DAC treatment reduced tumor growth *in vivo*. The effects of TS603 (left) were superior to that of TS667 (right). At least 10 mice (20 tumors) were used. B, DAC induces GFAP expression *in vivo*. Xenografts were sectioned and stained for GFAP. Results from vehicle or DAC treated tumors are shown.

To test the growth inhibitory effects of DAC *in vivo*, we treated TS603 and TS667 cells with 200 nM DAC for 7 days *in vitro*. We injected DAC or vehicle treated cells into the flanks of untreated SCID mice. Treatment with DAC markedly reduced the growth of TS603 xenografts by >90%. On the contrary, growth was more modestly impaired in the IDH1-wild-type TS667 glioma xenografts when compared to the IDH1-mutant expressing TS603 glioma xenografts (Fig. 3A). DAC treatment induced strong GFAP expression in IDH mutant TS603 cells but not in IDH wild-type TS667 cells (Fig. 3B).

### Low dose DAC inhibits genome-wide methylation and induces expression of genes associated with glial differentiation

**Figure 4:**
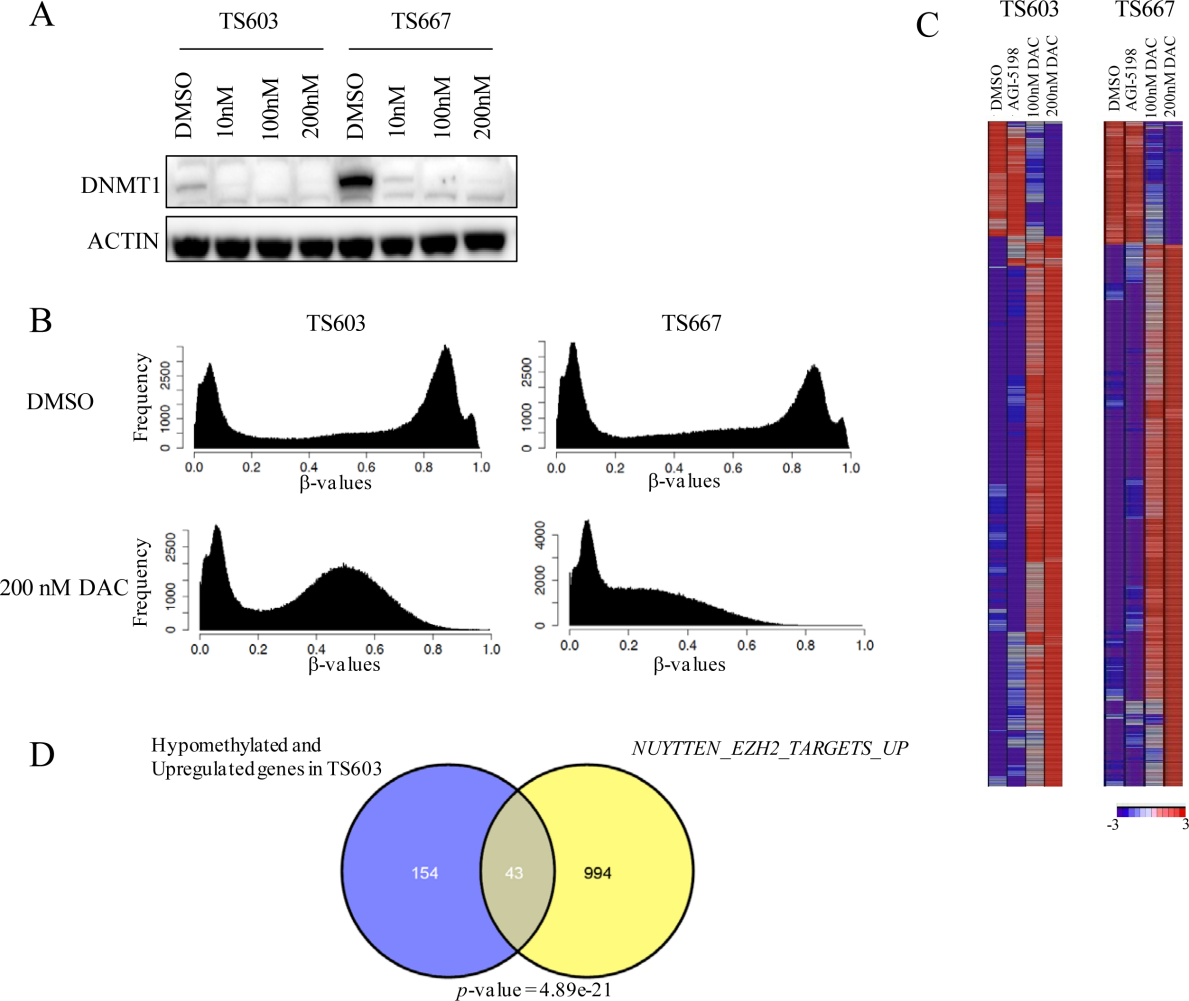
Decitabine reverses genome-wide DNA methylation and induces expression of genes associated with differentiation in IDH mutant glioma cells A, Low dose DAC inhibits DNMT1. Results from western blot shown. B, DAC treatment results in loss of DNA methylation. DNA methylome analysis of TS603 and TS667 cells following DAC treatment (200nM) is shown. Results from the Illumina HumanMethylation450 array. C, Gene expression changes following DAC treatment (200nM). Results from Affymetrix gene expression arrays. Most significantly altered genes following 200nM DAC are shown. D, Significant concordance between demethylated and upregulated genes and polycomb targets. Venn diagram showing overlap between the gene sets. P value (hypergeometric) is shown.

We evaluated the biochemical efficacy of DAC at low doses by assessing DNMT1 protein depletion after treatment. At the doses used, no cell death occurred. Both 3- and 7-day transient treatments with 100 and 200 nM DAC depleted DNMT1 levels, whereas 10 nM dose resulted in diminished levels of DNMT1 in TS603 and TS667 glioma cells (Fig. 4A). Maximal depletion of DNMT1 was rapid and occurred as early as following a 3-day treatment.

To assess genome-wide changes in methylation levels, we treated TS603 and TS667 glioma cells with vehicle, 100 nM DAC, 200 nM DAC or 1μM AGI-5198 continuously for 2 weeks. DNA methylation was assessed using the Illumina Infinium HumanMethylation450 BeadChip. Overall, there was a significant reduction in the number of methylated loci in both TS603 and TS667 glioma cells, although the degree of hypomethylation was more pronounced in the IDH1-wild-type TS667 line (Fig. 4B). After a 2-week exposure to 200 nM DAC, 30,915 probes (corresponding to 9996 unique genes) were hypomethylated in IDH1 mutant TS603 glioma cells and 130,017 probes (corresponding to 17,182 unique genes) were hypomethylated in TS667 cells (Δβ value < -0.4) (Supplemental Table 1). In contrast, only 52 probes were hypomethylated at Δβ < -0.4 in TS603 line after a 2-week treatment with 1μM AGI-5198 (Supplemental Table 1). Therefore, low dose DAC was a potent inhibitor of DNMT1 and loss of DNA methylation.

In addition to genome-wide methylation, we also assessed gene expression changes using Affymetrix gene expression arrays following a 2-week treatment with DMSO, 100nM DAC, 200 nM DAC or AGI-5198. In TS603 glioma cells, 411 unique genes were upregulated and 82 genes were downregulated after DAC treatment (Supplemental Table 2). Compared with vehicle-treated TS603 cells, low dose transient DAC treatment resulted in the upregulation of a number of genes associated with glial-astrocytic differentiation, such as *GFAP* and *LGALS3* [15], whereas *NEUROD1*, a gene associated with neural stem cells, was downregulated (Fig. 4C). Some of the most significantly enriched pathways (BH-adjusted *p* < 0.05) among the upregulated genes included ECM-receptor interaction, cytokine-cytokine receptor interaction and vasculature development (Supplemental Table 3).

When compared to vehicle treated cells, in IDH wild-type TS667 glioma cells, 385 unique genes were upregulated and 78 genes were downregulated after DAC treatment. Overall, 110 genes were upregulated in both cell lines after 200 nM DAC treatment, and upregulation of *GFAP* and other glial differentiation markers was not evident in TS667 glioma cells (Supplemental Table 2).

We next integrated the DNA methylation and gene expression data to identify genes that increase their gene expression following loss of methylation. Compared to vehicle treated controls, 79% of all the upregulated genes in TS667 cells were hypomethylated. In TS603 glioma cells, 48% of all upregulated genes (197 genes) were also hypomethylated (Supplemental Table 4). Importantly, this group also included all the differentiation-associated genes. To gain further insight into the genes with increased expression following 200nM DAC treatment in TS603 cells, our gene set consisting of hypomethylated and upregulated genes were analyzed using the molecular signature database (MSigDB) and the degree of overlap with the c2 curated gene sets was calculated. These results showed highly significant enrichment of *NUYTTEN_EZH2_TARGETS_UP*, which includes genes up-regulated in PC3 cells following EZH2 knockdown [16], indicating that DAC treatment may erase methylation marks from Polycomb EZH2 marked loci and subsequently activate genes involved in differentiation (Fig. 4D). These data point to the re-expression of Polycomb controlled genes as playing an important role in how DAC reverses the effects of mutant IDH.

## DISCUSSION

Tumor-initiating cells are thought to contribute to relapse or tumor recurrence after treatment, therefore a major challenge is to discover agents or treatment paradigms that will target this stem-cell like subpopulation. Our findings demonstrate the efficacy of DNA demethylating agents in targeting glioma-initiating cells and highlight the differential response of IDH1 mutant versus IDH1 wild-type glioma cells to low doses of DAC. Transient exposure to nanomolar concentrations of DAC induces the differentiation of the IDH1 R132H, but not IDH1 wild-type glioma-initiating cells. Importantly, exposure to nanomolar doses of DAC has long-lasting effects on continued differentiation, long after the demethylating agent is removed and DNMT1 levels are restored. This may be explained by the fact that exposure to DNA demethylating agents removes the DNA methylation marks at the promoters of glial differentiation genes such as *GFAP* and may also relax the formation of heterochromatin at the promoters of differentiation genes leading to their upregulation. In conjunction with increased differentiation in IDH1 R132H glioma cells, DAC also exerts its antitumor effects by reducing the tumorigenic potential of IDH1-mutant cells by decreasing colony formation *in vitro* and xenograft growth *in vivo*. It remains to be seen whether DNA demethylating agents can sensitize glioma-initiating cells to other cytotoxic or targeted drugs, as has been suggested in lung and ovarian cancers [17, 18]. In our initial studies, we have not observed sensitization of IDH1-mutant glioma cells to IDH1 R132H specific inhibitor, AGI-5198 following DAC exposure; however, further studies are needed.

Our studies also indicate that growth of IDH1 wild-type glioma cells are reduced following transient treatment with DAC, albeit through alternate pathways. The distinct gene expression program and the lack of differentiation phenotype observed in IDH1-wild-type cells suggest a different mechanism of action induced by DAC. Given the vast heterogeneity of glioma, a broader investigation of the differential therapeutic responses of glioma subgroups to transient low doses of DNA demethylating agents will shed light onto the therapeutic benefit of these agents as either mono- or combination therapies. Importantly, a companion paper by Greg Riggin's group at Johns Hopkins shows analygous results using a separately derived IDH mutant GIC line [19]. Together, our studies highlight the therapeutic potential of DNMT inhibitors for treatment of IDH mutant tumors.

The use of DAC to target mutant IDH dependent pathobiology is tantalizing. DAC is already FDA approved and penetrates the CNS very well. In our preclinical models, we show that using DAC can effectively reverse the pathologic DNA methylation induced by mutant IDH. Based on our data, we hypothesize that DAC may be useful for other tumor types, such as leukemias and chondrosarcomas, which have IDH mutation.

In summary, our findings highlight the utility of DNA demethylating agents in the management of glioma, a highly malignant disease with dismal prognosis. The ability to induce a sustained differentiation phenotype in the tumor-initiating subpopulation of IDH1 R132H gliomas may provide a promising therapeutic window of opportunity for tumors with this mutation.

## METHODS

### Generation of patient derived glioma-initiating cells and drug treatment

IDH1-R132H TS603 and IDH1-wild-type TS667 glioma spheres were derived from patients undergoing tumor resection at Memorial Sloan-Kettering Cancer Center (MSKCC). Tumors were obtained in accordance with Institutional Review Board policies at MSKCC. 10 nM, 100 nM, 200 nM Decitabine (Sigma-Aldrich, St. Louis, MO), 1 μM of AGI-5198 IDH1 R132H specific inhibitor (Xcess Bio) or DMSO were added to the media. Decitabine was added every 24 hours with daily medium change and AGI-5198 was added every other day with medium change.

### Soft agar assays

Cells were treated with decitabine for 3- or 7- days before plating for soft agar. 100,000 cells were plated in complete Neurocult media with growth supplements (Stem Cell Technologies) into 6-well plates containing 0.65% top and bottom agar. Cells were plated in the middle layer in complete Neurocult media containing 0.40% agar. Cells were treated with or without AGI-5198 for 3-4 weeks, and media was refreshed every 2 days. After 2-4 weeks, colonies were stained with 0.0005% crystal violet and quantified using a Gelcount colony counter (Oxford Optronix).

### Xenografts

TS603 and TS667 glioma cells were pretreated with 200 nM decitabine or DMSO for 7 days. After harvesting, 1x10^6^ cells were injected subcutaneously (100 μl volume, equal parts Matrigel and media) into both flanks of 5-6 week old female SCID mice. Once tumors researched a palpable size, tumor size was measured every 3-4 days by a caliper. Protocols for all treatments were approved by the Institutional Animal Care and Use Committee and strict guidelines were enforced.

### Western blot

Cells were lysed in CelLytic (Sigma) with protease inhibitors (Roche). Proteins were separated by SDS–PAGE, transferred to PVDF membrane (Millipore) and probed with the following primary antibodies: anti-IDH1 R132H (Dianova, DIA-H09), anti-DNMT1 (New England Biolabs, M0230S), anti-GFAP (Cell Signaling Tech, 2118S) and anti-β-actin (Sigma, A5316).

### Flow Cytometry

For single-color flow cytometry, 10^6^ cells were washed with ice-cold PBS, permeabilized and fixed using BD Cytoperm/Cytofix solution (BD, PharMingen), and incubated with anti-GFAP (1:200, BD Pharmingen) for 30 min at room temperature. Cells were washed with PBS and analyzed with FACScan flow cytometer (Becton Dickinson). Fluorescence-activated cell sorter data were analyzed using FLowJo Software (TreeStar).

### Immunohistochemistry

Paraffin-embedded sections of xenografts were deparaffinized. The sections were then stained with either hematoxylin and eosin (H&E), Ki-67 or GFAP. Detection was performed with the DAB Map kit (Ventana Medical Systems).

### Sample preparation

DNA from DMSO, DAC or IDH1 inhibitor treated cells were extracted with the DNeasy Blood and Tissue Kit (Qiagen) and RNA was isolated with RNeasy Plus Mini Kit according to the manufacturer's directions.

### Genomic analysis

Expression analysis was performed using the Affymetrix U133 2.0 microarray (Affymetrix). Genome-wide methylation analysis was performed using the Infinium HumanMethylation450 bead array (Illumina). Processing of the arrays was per the manufacturers' protocol. Methylation data were extracted using GenomeStudio software (Illumina). Methylation values for each site are expressed as a beta (β) value, representing a continuous measurement from 0 (completely unmethylated) to 1 (completely methylated). This value is based on following calculation: β value = (signal intensity of methylation-detection probe)/(signal intensity of methylation- detection probe + signal intensity of non-methylation detection).

### Data analysis

For methylation analysis, Illumina data were imported into Partek software. β values for 200 nM DAC or 1uM AGI-5198 treated cells were substracted from β values for DMSO treated cells. Any probe with Δβ < -0.4 were considered to be hypomethylated following treatment. For gene expression analysis, microarrays were RMA normalized and fold changes were calculated by subtracting the log intensity values of 200 nM DAC or 1uM AGI-5198 from DMSO treated cells, and probes with absolute fold changes > 1.2 were considered to be differentially expressed. DAVID was used to identify significantly overrepresented pathways [20]. For gene set analysis, hypomethylated and upregulated genes were input into the molecular signature database (MSigDB) and statistical significance of overlaps were calculated between our gene sets and the C2 (curated gene sets) library from MSigDB.

### 2-HG analysis

2HG levels were determined by mass spectrometry as previously described [6].

## Supplemental Tables


